# *Ex Vivo* Evaluation of Egg Yolk IgY Degradation in Chicken Gastrointestinal Tract

**DOI:** 10.3389/fimmu.2021.746831

**Published:** 2021-09-21

**Authors:** Huiwen Wang, Ximin Zeng, Jun Lin

**Affiliations:** Department of Animal Science, The University of Tennessee, Knoxville, TN, United States

**Keywords:** egg yolk antibody, passive immunization, IgY stability, chicken gizzard, gastrointestinal digestion

## Abstract

Egg yolk antibody (immunoglobulin Y, IgY), due to its unique features (e.g., cost-effectiveness for mass production), is emerging as a promising passive immune agent and alternative to antibiotics to combat infectious diseases, particularly in livestock. Oral administration of egg yolk IgY is the most common and convenient route that has been extensively investigated for controlling enteric pathogens. However, the *in vivo* stability of egg yolk IgY in the gastrointestinal (GI) tract, a critical issue for the success of this approach, still has not been clearly elucidated. Our recent study showed instability of orally administered egg yolk IgY in chicken GI tract, as demonstrated by both *in vivo* and *ex vivo* evidence. To better understand the magnitude and dynamics of instability of egg yolk IgY *in vivo*, in this study, we conducted comprehensive *ex vivo* analyses by spiking hyperimmune egg yolk IgY in fresh GI contents collected from five broilers at each sampling age (2, 4, or 6 weeks). The pH in gizzard slightly increased with age from 2.4 to 3.0, while the pH in the small intestine was around 5.8. ELISA analysis indicated that a short time of treatment (30 or 60 min) of IgY with the gizzard contents from the chickens at 2, 4, and 6 weeks of age greatly reduced specific IgY titer by over 8, 6, and 5 log_2_ units, respectively, when compared with saline control. However, small intestine content only had a mild effect on egg yolk IgY, leading to 1 log_2_ unit of reduction in IgY titer upon 30 min of treatment. Consistent with these findings, SDS-PAGE and immunoblotting analyses provided direct evidence demonstrating that egg yolk IgY could be drastically degraded to undetectable level in gizzard content upon as short as 5 min of treatment; however, the IgY was only slightly degraded in small intestine content. Immunoblotting also showed that treatment of IgY with HCl (pH 3.0) for 60 min did not affect its integrity at all, further supporting the enzymatic degradation of IgY in gizzard. Collectively, egg yolk IgY could be substantially degraded in chicken gizzard, highly warranting the development of effective approaches, such as encapsulation, for the controlled release and protection of orally administered egg yolk IgY in livestock.

## Introduction

Chicken immunoglobulin Y (IgY) is the functional equivalent of IgG in mammals and can be transferred from serum to yolk during egg formation. Egg yolk contains a large quantity of IgY that can confer passive immunity for chicks against pathogens, either at embryonic or post-hatching stage (1). Immunization of hens with a particular antigen could yield a large amount of specific egg yolk IgY that has potential applications in human and veterinary medicine (2). In particular, as an emerging alternative to antibiotics, egg yolk IgY has drawn considerable research interest in recent decades due to several unique features (2). First, a laying hen is regarded as a cost-efficient “bioreactor” that can produce over 22.5 g of egg yolk IgY yearly with 2%–10% being antigen-specific (3). In addition, collecting egg yolk—a non-invasive practice—is convenient and also favorable from an animal welfare perspective (3). Finally, egg yolk IgY is quite stable in a wide pH range (3.5–11) and under high temperatures (up to 70°C), making it feasible for storage and processing as a feed supplement (2).

Due to these enticing features of egg yolk IgY, numerous studies have been conducted to develop specific egg yolk IgY for the control of microbial infections in humans and animals (1). In these studies, egg yolk IgY was administrated to animal hosts *via* different routes, depending on the infection sites of targeted pathogens. Oral administration through feed or drinking water is the most common and convenient approach, especially against enteric pathogens (1). However, the *in vivo* stability and bioavailability of administered egg yolk IgY in the gastrointestinal (GI) tract have not been examined in most previous studies and are still largely unknown to date. Addressing this issue is critical for appropriately assessing the efficacy of orally administrated egg yolk IgY and for developing effective egg yolk IgY-based passive immune intervention strategies. Limited *in vitro* data are available concerning the stability of IgY in response to pepsin (a gastric protease), trypsin (a protease in the small intestine), and low pH alone (4–6). It has been reported that the neutralizing activity of IgY against rotavirus was totally lost in a pepsin solution (pH 2) but largely retained in a trypsin solution (pH 8) (5). In addition, IgY was fairly stable under the pH ranging from 3.5 to 11 (6). However, these *in vitro* studies could not fully reflect *in vivo* conditions of the GI tract. Therefore, a well-controlled *ex vivo* system to examine the fate of egg yolk IgY in GI tract is highly needed.

In a recent chicken trial (7), we have observed that orally administered hyperimmune egg yolk IgY was not stable in chicken GI tract, likely due to significant enzymatic degradation in chicken gizzard, which was supported by enzyme-linked immunosorbent assay (ELISA) analysis of specific IgY in both *in vivo* and *ex vivo* samples. In this study, to better understand the magnitude and dynamics of instability of egg yolk IgY in the GI tract and obtain direct evidence of IgY degradation, fresh GI contents were collected from five individual chickens at each sampling age (2, 4, or 6 weeks), slightly processed with saline, and subsequently subjected to comprehensive *ex vivo* analyses using multiple approaches [i.e., ELISA, sodium dodecyl sulfate-polyacrylamide gel electrophoresis (SDS-PAGE), and immunoblotting].

## Materials and Methods

### Collection of Chicken GI Content Samples

The chicken trial performed in this study was approved by the Institutional Animal Care and Use Committee at The University of Tennessee, Knoxville, with protocol number 1387-0219. Briefly, 15 newly hatched broiler chicks (Hubbard Efficiency Plus) were fed with regular feed for 6 weeks. At 2, 4, or 6 weeks of age, five chickens were randomly selected and euthanized. The pH in gizzard and small intestine (the junction site between the duodenum and jejunum) was measured on site using a pH spear tester (Thermo Fisher, MA, USA). Subsequently, the fresh GI contents (5 ml per sample) were collected using clean stainless steel laboratory scoops, transferred into 15 ml sterile plastic tubes, stored on ice, and immediately subjected to downstream work as detailed below.

### *Ex Vivo* IgY Stability Assays

The *ex vivo* IgY stability assays were performed as described previously (5, 7) with modifications. Because supplementation of lyophilized egg yolk powder in feed is the most practical and widely adopted approach for oral administration of egg yolk IgY to control microbial infections in food animals (1), the lyophilized hyperimmune egg yolk powder produced in our recent study (7) was utilized for *ex vivo* assays to assess IgY stability in GI contents. The egg yolk powder was reconstituted in saline at the ratio of 1:3 (w/w), followed by centrifugation at 13,000*g* for 2 min. The supernatant containing egg yolk IgY was used as stock to spike various GI samples. Each of the fresh gizzard and small intestine content samples was gently mixed with same volume of sterile saline and centrifuged at 5,000*g* for 5 min. The supernatant was kept and used as corresponding GI content matrix system for *ex vivo* IgY stability assay. The supernatant was measured for pH and spiked with 5% (v/v) of the egg yolk IgY solution, while saline or HCl (1 mM, pH 3.0) served as a control. The mixtures were incubated at 42°C (chicken body temperature) for different lengths of time. Subsequently, the mixtures were subjected to ELISA or SDS-PAGE analysis. If needed, some mixtures containing gizzard content or HCl were neutralized to pH 7.0 using less than 2% volume of NaOH upon completion of treatment.

### Enzyme-Linked Immunosorbent Assay

Indirect ELISA was conducted as described previously (8) to determine the titers of specific egg yolk IgY in the mixture after specific treatment. Briefly, the ELISA plates (Nunc MaxiSorp, Thermo Fisher, MA, USA) were coated with 100 μl of immunogen [keyhole limpet hemocyanin–enterobactin conjugate (8), 300 ng/ml] in coating buffer (bicarbonate/carbonate, pH 9.6) overnight at room temperature. The coated plates were blocked with blocking buffer (PBS + 0.05% Tween 20 + 5% skim milk) at room temperature for 1 h. Subsequently, the aforementioned samples under various treatments were two-fold serially diluted in blocking buffer and incubated with the coated plates at room temperature for 1 h. Following four times washing with PBS containing 0.05% of Tween 20, 100 μl of the horseradish peroxidase-conjugated goat anti-chicken IgY secondary antibody (SeraCare, MA, USA; 2,000-fold diluted in blocking buffer) was added in each well, and the plates were incubated at room temperature for 1 h. After four times washing with the washing buffer, the plates were finally developed using 100 μl of ABTS^®^ One-component Peroxidase Substrate (SeraCare), and the reaction was stopped after 30 min by adding 100 μl of stopping solution (1% SDS). The absorbance was measured at optical density of 405 nm (OD_405_) using a microplate reader (BioTek Instruments, VT, USA) and data were collected using Gen5 software (BioTek Instruments). The wells without the addition of primary antibody served as a blank control. Endpoint titer was defined as the last dilution at which OD_405_ of sample wells exceeded the cutoff value (0.1). Duplicate measurements were performed for each sample.

### Purification of IgY From Egg Yolk

The hyperimmune egg yolk generated in our recent study (9) was used for IgY purification following a published protocol (10) with slight modifications. Briefly, one volume of egg yolk was diluted with four volumes of PBS and then mixed with 3.5% (w/v) of polyethylene glycol 6000 (PEG 6000, Fisher Scientific, MA, USA) for lipid removal. After 20 min of incubation at room temperature, the mixture was centrifuged at 5,000*g* for 20 min. Subsequently, the supernatant was added with 12% (w/v) of PEG 6000 for IgY precipitation and the mixture was incubated at room temperature for 10 min. Following centrifugation at 5,000*g* for 20 min, the precipitate was dissolved in PBS and added with 12% (w/v) of PEG 6000 again for another round of IgY precipitation. After centrifugation, the precipitate was dissolved in a small volume of PBS, added with equal volume of precooled (−20°C) ethanol, and centrifuged at 5,000*g* and 4°C for 20 min to remove PEG 6000. Lastly, the precipitate containing a high proportion of IgY was dissolved in a small volume of PBS and the solution was dialyzed against PBS. Initial egg yolk, the supernatant and precipitate fractions obtained from the centrifugation of 12% PEG 6000 mixture, and the final dialyzed IgY extract were subjected to SDS-PAGE analysis.

### Sodium Dodecyl Sulfate-Polyacrylamide Gel Electrophoresis and Immunoblotting

All *ex vivo* assay samples were subjected to SDS-PAGE analysis [12% (w/v) polyacrylamide gel, Bio-Rad, CA, USA] and stained with Coomassie blue R-250 dye (Thermo Fisher, USA). For immunoblotting, the proteins on polyacrylamide gel were electrotransferred to a nitrocellulose membrane, which was subsequently blocked in blocking buffer (PBS + 0.05% Tween 20 + 5% skim milk) at 4°C overnight. Then the membrane was incubated with goat anti-chicken IgY-horseradish peroxidase (SeraCare, 5,000-fold diluted in blocking buffer) at room temperature for 1 h. After the washing procedure (PBS + 0.05% Tween 20), the membrane was developed using SuperSignal™ West Pico PLUS Chemiluminescent Substrate (Thermo Fisher, USA).

### Statistical Analysis

Statistical analyses were conducted using Mann–Whitney test in GraphPad Prism 8. Data were presented as mean ± standard deviation (SD). A probability level of *P <*0.05 was considered as a statistically significant difference.

## Results

### The pH in the Chicken GI Tract

As shown in **Table 1**, the pH in gizzard slightly increased with age, which was 2.42, 2.90, and 2.96 at 2, 4, and 6 weeks of age, respectively. There was no significant difference among the *in vivo* pH at these three time points. After dilution of the gizzard content using saline, the supernatant obtained for *ex vivo* assays had slightly elevated pH, which was approximately 0.57 higher than the corresponding original gizzard content (**Table 1**). However, as expected, the small intestine had approximate pH of 5.84 at 6 weeks of age; the pH barely changed in the corresponding supernatant used for *ex vivo* assay upon slight dilution with saline (**Table 1**).

**Table 1:** pH in the chicken GI tract (*in vivo*) and the corresponding *ex vivo* samples at different ages.

	Gizzard^a^			Small intestine^a^
	Week 2	Week 4	Week 6	Week 6
*In vivo*	2.42 ± 0.28	2.90 ± 0.54	2.96 ± 0.79	5.84 ± 0.12
*Ex vivo*	3.06 ± 0.28	3.23 ± 0.61	3.71 ± 0.56	5.75 ± 0.10

a The data are presented as mean ± SD of five individual chicken samples.

### Drastic Reduction of Specific Egg Yolk IgY Titer in Gizzard Content

**Figure 1:**
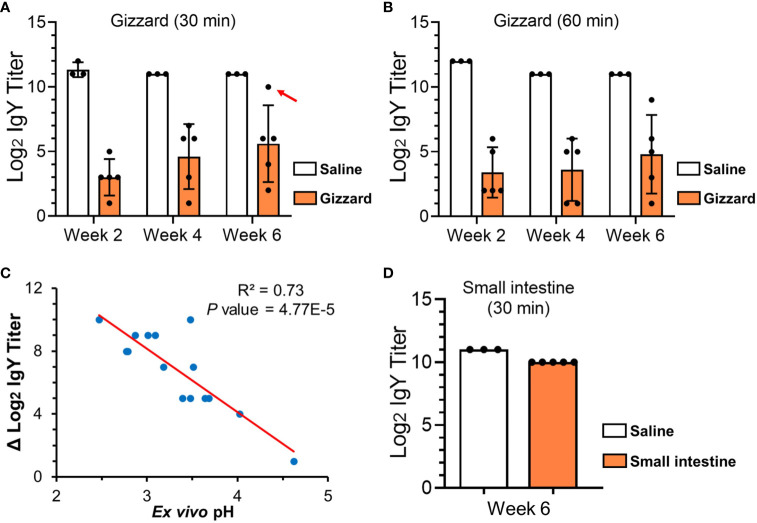
ELISA titers of specific egg yolk IgY in the GI samples collected from chickens at different ages. **(A)** Specific egg yolk IgY titers upon treatment in saline (control) or gizzard contents collected at 2, 4, and 6 weeks of age at 42°C for 30 min. The dot indicated by a red arrow is an outlier sample with the highest *ex vivo* pH of 4.62. **(B)** Specific egg yolk IgY titers upon treatment in saline (control) or gizzard contents at 2, 4, and 6 weeks of age at 42°C for 60 min. **(C)** Scatter plot showing an inverse correlation between *ex vivo* pH in the assays and the reduction of specific IgY titer upon 30 min of treatment in gizzard content. The dots represent each of the 15 gizzard samples at different ages. *P*-value of 4.77E-5 indicates a significant linear correlation between the two variables. **(D)** Specific egg yolk IgY titers upon treatment in saline (control) or small intestine contents at 6 weeks of age at 42°C for 30 min. In the bar plots above, data are presented as mean ± SD of measurements from five chicken samples or three saline controls at each specific time point.

With as short as 30 min of incubation with the gizzard content, the specific IgY titers in egg yolk were significantly reduced by over 8, 6, and 5 log_2_ units at 2, 4, and 6 weeks of age, respectively, when compared with saline control (**Figure 1A**). After an additional 30 min of treatment, the specific IgY titers at the three time points were further lowered by an average of 0.5 log_2_ unit (**Figure 1B**). Because escalation of pH in gizzard with age (**Table 1**) appeared to be associated with the magnitude of reduction in specific IgY titers (**Figures 1A, B**), further correlation analysis was performed and revealed that there was a significant inverse correlation (*P* < 0.01) between *ex vivo* sample pH and the reduction of specific IgY titer in response to 30 min of treatment in gizzard content (**Figure 1C**).

### IgY Titer Slightly Decreased Upon Treatment With Small Intestine Content

As shown in **Figure 1D**, incubation of egg yolk IgY with small intestine content obtained from the chickens at 6 weeks of age only led to 1 log_2_ unit (two-fold) of reduction when compared with saline control.

### Egg Yolk IgY Was Greatly Degraded in Gizzard Content

**Figure 2:**
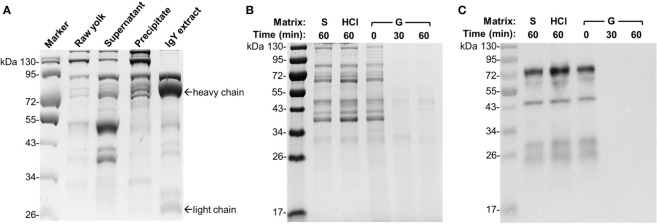
Integrity of egg yolk IgY in response to the treatment with HCl or gizzard content. **(A)** SDS-PAGE analysis of stepwise purification of IgY from egg yolk. Raw yolk: the egg yolk prior to IgY purification; supernatant and precipitate: the fractions obtained following centrifugation of 12% PEG 6000-treated egg yolk mixture; IgY extract: the purified IgY after final dialysis. **(B)** SDS-PAGE and **(C)** immunoblotting analyses of the integrity of egg yolk IgY in response to the treatment with HCl (1 mM, pH 3.0) or a representative gizzard content (pH 3.0) at 42°C for the indicated length of time. The reconstituted hyperimmune egg yolk powder was used as stock to spike different matrixes as described in the *Materials and Methods*. The far left lane is a protein marker. Abbreviation of the matrix system for treating egg yolk IgY: S, saline; G, gizzard content.

The above dynamic assessment of IgY stability in gizzard content confirmed our previous *in vivo* and *ex vivo* findings (7) and suggested enzymatic degradation of IgY in gizzard. To obtain direct evidence of IgY degradation, we subsequently conducted SDS-PAGE and immunoblotting assays on the egg yolk IgY upon different treatments. We first performed stepwise purification of IgY from egg yolk powder to better understand the profile of IgY on SDS-PAGE. As shown in **Figure 2A**, the high-purity IgY extract was obtained after stepwise purification and displayed characteristic bands of heavy chain and light chain as reported previously (11).

To compare the effect of gizzard content or low pH on the integrity of egg yolk IgY, the egg yolk was spiked in a representative gizzard content sample (2 weeks of age, pH 3.0) or HCl solution (pH 3.0). The egg yolk treated in the matrixes of control saline, HCl for 60 min, and the gizzard content for 0 min consistently displayed multiple protein bands with similar pattern (**Figure 2B**). However, both 30 and 60 min treatments of egg yolk in gizzard content led to all bands barely visualized (**Figure 2B**). Consistent with this finding, immunoblotting using specific anti-IgY antibodies clearly indicated that the IgY was undetected after 30 or 60 min of treatment in gizzard content, while its integrity was still maintained well in the matrix of control saline or HCl upon 60 min of treatment (**Figure 2C**). These data are also consistent with previous ELISA analysis of IgY titer upon acid treatment (7) and provide further direct evidence demonstrating that acidic condition alone (e.g., pH 3.0) did not alter the IgY integrity.

### Potent but Varied Degradation of Egg Yolk IgY in Individual Gizzard Content Samples

**Figure 3:**
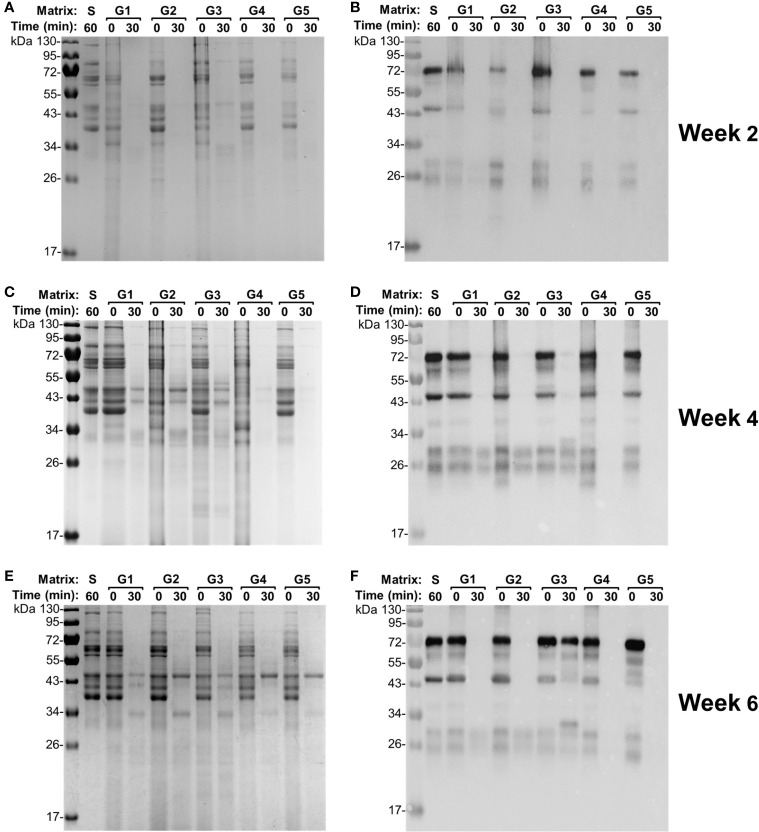
Integrity of egg yolk IgY in response to the treatment with the gizzard content collected from each individual chicken. **(A)** SDS-PAGE and **(B)** immunoblotting analyses of the integrity of egg yolk IgY in response to the treatment with each individual gizzard content collected at 2 weeks of age at 42°C for 0 or 30 min. The reconstituted hyperimmune egg yolk powder was used as stock to spike the gizzard content matrixes as described in the *Materials and Methods*. The far left lane is a protein marker. Abbreviation of the matrix system for treating egg yolk IgY: S, saline; G1–G5, gizzard contents of five individual chickens at 2 weeks of age (indicated on the right). Likewise, **(C)** SDS-PAGE and **(D)** immunoblotting analyses of the integrity of egg yolk IgY upon treatment in each individual gizzard content collected at 4 weeks of age; **(E)** SDS-PAGE and **(F)** immunoblotting analyses of the integrity of egg yolk IgY upon treatment in each individual gizzard content collected at 6 weeks of age.

To further examine the degradation capacity of individual gizzard contents collected from the chickens at different ages, each gizzard sample spiked with egg yolk was subjected to 30 min of incubation at 42°C followed by SDS-PAGE and immunoblotting analyses (**Figure 3**). Incubation of egg yolk with the five gizzard samples from the chickens at 2 weeks of age made all bands almost fade away when compared with similar multiband pattern observed in control saline and 0 min of treatment (**Figure 3A**). Consistent with this SDS-PAGE result, immunoblotting using specific anti-IgY antibodies showed that the IgY was undetected after 30 min of treatment in each gizzard content when compared with the characteristic IgY bands in the matrix of control saline (**Figure 3B**). A similar finding—potent degradation of egg yolk IgY in gizzard content upon 30 min of treatment—was also observed in almost all the samples collected from the chickens at 4 weeks (**Figures 3C, D**) and 6 weeks of age (**Figures 3E, F**). However, incubation of egg yolk IgY in one gizzard content collected from a 6-week-old chicken (G3) for 30 min did not cause significant IgY degradation as shown by immunoblotting analysis (**Figure 3F**); notably, this gizzard content was an outlier because this *ex vivo* sample had much higher pH (4.62) than other samples (**Table 1**) and only led to 1 log_2_ unit of reduction in IgY titer when compared with saline control (the dot indicated by a red arrow in **Figure 1A**).

### Time Course Analysis of IgY Degradation in Gizzard Content

**Figure 4:**
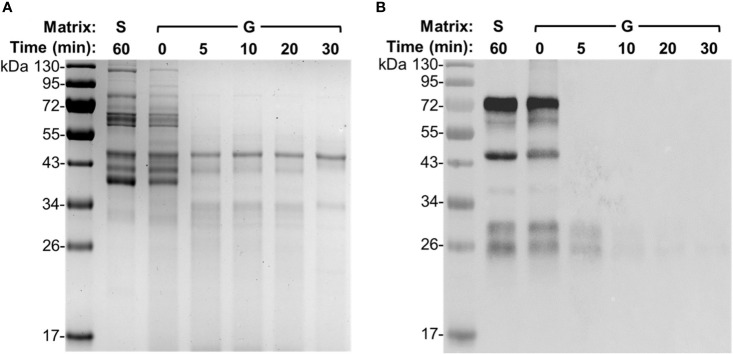
Degradation dynamics of egg yolk IgY in gizzard content. **(A)** SDS-PAGE and **(B)** immunoblotting analyses of the integrity of egg yolk IgY in response to the treatment with a representative gizzard content at 42°C for the indicated length of time. The reconstituted hyperimmune egg yolk powder was used as stock to spike the gizzard content matrixes as described in the *Materials and Methods*. The far left lane is a protein marker. Abbreviation of the matrix system for treating egg yolk IgY: S, saline; G, gizzard content.

To further determine the efficiency and capability of gizzard content to degrade egg yolk IgY, time course analysis was performed using a gizzard content sample at 6 weeks of age with incubation time ranging from 0 to 30 min. As shown in SDS-PAGE (**Figure 4A**), the pattern of protein bands at the beginning (0 min) of the gizzard treatment was very similar to that in saline with only subtle reduction in the intensity of most bands. Nevertheless, most bands were not visualized after only 5 min of treatment; there was no further notable change afterwards (i.e., at 10, 20, and 30 min) (**Figure 4A**). Consistently, immunoblotting showed that the IgY bands were almost undetected after as short as 5 min of treatment, while the characteristic IgY bands in both saline control and 0 min of sample were vividly detected by IgY-specific antibodies (**Figure 4B**).

### Small Intestine Content Displayed IgY-Degrading Capacity but With Less Magnitude Than Gizzard

**Figure 5:**
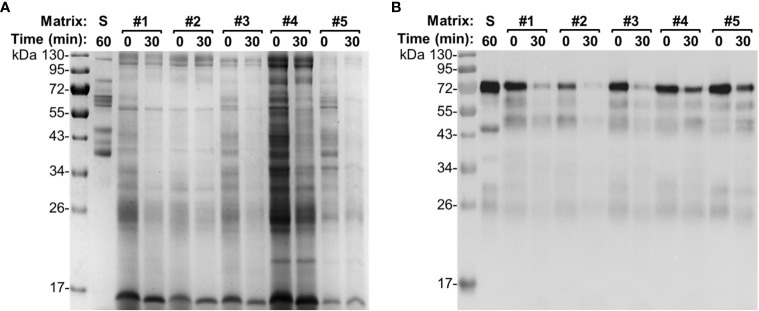
Integrity of egg yolk IgY in response to the treatment with small intestine content collected from each individual chicken at 6 weeks of age. **(A)** SDS-PAGE and **(B)** immunoblotting analyses of the integrity of egg yolk IgY in response to the treatment with each individual small intestine content collected at 6 weeks of age at 42°C for 0 or 30 min. The reconstituted hyperimmune egg yolk powder was used as stock to spike the small intestine content matrixes as described in the *Materials and Methods*. The far left lane is a protein marker. Abbreviation of the matrix system for treating egg yolk IgY: S, saline; #1–#5, small intestine contents of five individual chickens at 6 weeks of age.

In our recent study (7), the pooled small intestine content did not affect the titer of specific IgY with up to 2.5 h of treatment, suggesting weak or lack of IgY-degrading capability of small intestine content. In this study, using SDS-PAGE and immunoblotting analyses, we further evaluated the IgY-degrading capacity of each small intestine content from five 6-week-old chickens. Unlike what was observed for the 30-min incubation of egg yolk IgY with gizzard content (**Figures 2**, **3**), the 30-min incubation of egg yolk IgY with small intestine content led to moderate reduction in the intensity of various protein bands on SDS-PAGE (**Figure 5A**). Nevertheless, immunoblotting clearly indicated that majority of spiked IgY was degraded in the first three samples while much less was degraded in samples #4 and #5 (**Figure 5B**).

## Discussion

In this study, we collected the contents from chicken gizzard and upper region of the small intestine (the junction between the duodenum and jejunum) for *ex vivo* analysis of egg yolk IgY stability based on following three major reasons. First, gizzard and small intestine are recognized as the major digestive organs, which may directly degrade egg yolk IgY upon oral administration due to presence of various proteases (12). Pepsin, a gizzard protease whose function strictly relies on acidic condition resulting from secretion of hydrochloric acid (average pH 3.0), plays a critical role in the first line of feed digestion during feed retention in poultry (12). Other digestive enzymes, trypsin in particular, are produced in the pancreas and then released into the upper region of the small intestine for further feed digestion (13). Second, a panel of significant enteric pathogens, such as *Campylobacter jejuni* and *Salmonella enterica* serotype Typhimurium, colonize predominantly in the lower region of the intestine (e.g., cecum) (14). Therefore, orally administered egg yolk IgY should take a long journey—up to 80 min of retention time in gizzard and approximately 30 min of retention time in the upper region of the small intestine—to reach the desired target site for pathogen control (15, 16). During this long journey, egg yolk IgY may encounter a panel of harsh conditions that potentially affect its stability, such as low pH, proteases, and free radicals (12). Consequently, examination of the stability of egg yolk IgY in the upper GI tract is a critical issue for the success of using orally administrated IgY for passive immune intervention. Third, chicken crop, an organ prior to gizzard, is for transient storage and moisturization of ingested feed with internal pH above 6.0 (12). It is well recognized that crop does not have digestive functions and there are no secreted digestive enzymes in crop (12). Therefore, egg yolk IgY is expected to be stable in crop and we did not include crop content for *ex vivo* analysis here.

In this study, we have taken into careful consideration several critical parameters in *ex vivo* assay to better mimic *in vivo* conditions, with emphasis on reaction time and pH. Under physiological condition, the retention of feed in chicken gizzard ranges from 30 to 80 min, while the retention time in the upper small intestine is approximately 30 min (15, 16). Based on these facts, we examined the stability of egg yolk IgY in gizzard content for up to 60 min or in the small intestine content for 30 min. Since titer reductions of the specific IgY in gizzard content were comparable between 30 and 60 min of treatments (**Figures 1A, B**), in the subsequent SDS-PAGE and immunoblotting analyses, we only examined the integrity of IgY upon 30 min of treatment with each of the 15 gizzard content samples. With respect to pH measurement, the pH meter with a thin probe enabled us to accurately determine the *in vivo* pH in the GI tract. The GI content was slightly diluted with saline to minimize any artificial effects for *ex vivo* assay. Despite a slight increase of pH by an average of 0.57 in saline-processed gizzard contents when compared with the corresponding original *in vivo* contents (**Table 1**), pepsin in gizzard content should still remain over 50% of its maximal enzymatic activity because the pH of all but one saline-processed gizzard contents was still below 4.0 (17). The only outlier of gizzard content had pH 4.62 (described in the *Results*), which may be the major factor leading to a slight reduction (two-fold) in IgY titer (indicated by an arrow in **Figure 1A**) and substantial residual IgY detected in immunoblotting (G3 in **Figure 3F**) upon 30 min of treatment in this gizzard content. Together, the slightly increased pH in the saline-processed gizzard contents used for *ex vivo* assays here might underestimate the capability of gizzard content to degrade IgY *in vivo* but would not affect the conclusion drawn from this study. Finally, despite compelling evidence here showing the instability of egg yolk IgY in the GI tract, we would like to mention that this study is still limited by using the broilers raised in an animal facility. Examination of the GI contents collected from the birds raised in a poultry production setting with interflock replication is highly warranted in future studies.

Egg yolk IgY is increasingly recognized as a promising passive immune agent for the control of various microbial infections (1). To control enteric pathogens or toxins, egg yolk IgY has been commonly administrated through feed or drinking water to different animals (18). These studies have yielded varied successes, such as inhibiting microbial infections or improving growth performance (3, 18). However, the *in vivo* level and stability of orally administered egg yolk IgY have never been assessed in numerous studies conducted by other groups, which is surprising because egg yolk IgY may be undermined when passing through the GI tract of an animal, a destructive environment for proteins. To fill this significant knowledge gap, in this study, we performed delicate *ex vivo* assays to simulate the effect of the GI tract on egg yolk IgY. The findings from this study, for the first time, provided direct and compelling evidence demonstrating that egg yolk IgY was dramatically degraded shortly after its exposure to gizzard content and could also be subjected to moderate degradation by small intestine content. However, it is interesting that numerous studies showed the efficacy of orally administered egg yolk IgY against different infectious agents (1, 2). Notably, none of the previous studies from other research groups examined *in vivo* level and stability of the orally administrated egg yolk IgY when performing challenge trial, greatly impeding us to properly link the phenotype (e.g., reduced pathogen load or clinical signs) to the antimicrobial effect resulting from orally administered egg yolk IgY. Thus, the findings from previous studies need to be revisited and confirmed in conjunction with examination of the *in vivo* stability and level of the specific egg yolk IgY administered *via* the oral route.

We expect that the findings from this study can also be extrapolated to other animals and even humans. For monogastric organisms (e.g., mouse, rabbit, swine, and human), pepsins are produced in their stomachs as the case in chicken gizzard. The low pH in these stomachs (1.5–4.0 in general) can fulfill the requirement of pepsins to exert degradation on various peptides and proteins (19–21). Ruminant (e.g., bovine and ovine) abomasum, equivalent to the stomach of monogastric animals in anatomy and function, also has low pH environment (usually 1.5–2.5) and pepsin for protein digestion (22, 23). Therefore, to successfully utilize hyperimmune egg yolk as a passive immune agent to control enteric pathogens, it is critical to develop cost-effective strategies to prevent undesired degradation of the IgY in the GI tract, particularly in the stomach, and facilitate the delivery of functional egg yolk IgY to a specific GI site.

Encapsulation of egg yolk IgY is a feasible strategy for protecting the IgY from degradation in the GI tract of food-producing animals. Various coating strategies and materials have been assessed for microencapsulation of the IgY, such as alginate/chitosan, multilayer polypeptides, methacrylic acid copolymer, or alginate/carrageenan microgels (24–27). These approaches, to different extents, prevented IgY activity loss and achieved controlled release of IgY in the simulated GI fluid matrix system (24–27). However, these past studies have some limitations. First, the encapsulation procedures used in these studies were lengthy and complicated. For instance, encapsulation with alginate/chitosan involved the purification of IgY from egg yolk, preparation of encapsulation medium, and loading IgY into the microcapsules using professional facilities, as well as filtration, rinse, and lyophilization of the microcapsules (25). Second, some of the encapsulation materials, such as methacrylic acid copolymer (24) or multilayer polypeptides (26), were expensive, potentially impeding practical application of this strategy in livestock. Finally and more importantly, only the *in vitro* system, i.e., the buffer supplemented with digestive enzymes, was used to evaluate the efficacy of encapsulation in IgY protection and release in these studies (24–27). Since there are complex factors present in the GI tract, it is critically important to perform *ex vivo* as well as *in vivo* studies to evaluate encapsulation strategy for protection and controlled release of IgY. Therefore, in future studies, cost-effectiveness, easiness, and *in vivo* assessment should be taken into special consideration when developing encapsulation methods for protection and controlled release of hyperimmune egg yolk IgY in the intestine of livestock.

## Data Availability Statement

The raw data supporting the conclusions of this article will be made available by the authors, without undue reservation.

## Ethics Statement

The animal study was reviewed and approved by the Institutional Animal Care and Use Committee at The University of Tennessee, Knoxville, with protocol number 1387-0219.

## Author Contributions

Conceptualization: HW, XZ, and JL. Methodology: HW, XZ, and JL. Investigation: HW, XZ, and JL. Data analysis: HW and JL. Manuscript writing: HW and JL. Project administration and funding acquisition: JL. All authors contributed to the article and approved the submitted version.

## Funding

This study was supported by the United States Department of Agriculture National Institute of Food and Agriculture (NIFA) Award No. 2018-67015-28295.

## Conflict of Interest

The authors declare that the research was conducted in the absence of any commercial or financial relationships that could be construed as a potential conflict of interest.

## Publisher’s Note

All claims expressed in this article are solely those of the authors and do not necessarily represent those of their affiliated organizations, or those of the publisher, the editors and the reviewers. Any product that may be evaluated in this article, or claim that may be made by its manufacturer, is not guaranteed or endorsed by the publisher.

## References

[1] Kovacs-Nolan J Mine Y. Egg Yolk Antibodies for Passive Immunity. Annu Rev Food Sci Technol (2012) 3:163–82. doi: 10.1146/annurev-food-022811-101137 22136128

[2] Pereira E van Tilburg M Florean E Guedes M. Egg Yolk Antibodies (IgY) and Their Applications in Human and Veterinary Health: A Review. Int Immunopharmacol (2019) 73:293–303. doi: 10.1016/j.intimp.2019.05.015 31128529 PMC7106195

[3] Xu Y Li X Jin L Zhen Y Lu Y Li S. Application of Chicken Egg Yolk Immunoglobulins in the Control of Terrestrial and Aquatic Animal Diseases: A Review. Biotechnol Adv (2011) 29(6):860–8. doi: 10.1016/j.biotechadv.2011.07.003 PMC7126572 21787857

[4] Jaradat ZW Marquardt RR. Studies on the Stability of Chicken IgY in Different Sugars, Complex Carbohydrates and Food Materials. Food Agr Immunol (2000) 12(4):263–72. doi: 10.1080/09540100020008137

[5] Hatta H Tsuda K Akachi S Kim M Yamamoto T Ebina T. Oral Passive Immunization Effect of Anti-Human Rotavirus IgY and Its Behavior Against Proteolytic Enzymes. Biosci Biotechnol Biochem (1993) 57(7):1077–181. doi: 10.1271/bbb.57.1077 7764069

[6] Shimizu M Nagashima H Sano K Hashimoto K Ozeki M Tsuda K. Molecular Stability of Chicken and Rabbit Immunoglobulin G. Biosci Biotechnol Biochem (1992) 56(2):270–4. doi: 10.1271/bbb.56.270 1368302

[7] Wang H Zeng X Cao L He Q Lin J. Passive Immunization of Chickens With Anti-Enterobactin Egg Yolk Powder for *Campylobacter* Control. Vaccines (2021) 9(6):569. doi: 10.3390/vaccines9060569 34205835 PMC8230082

[8] Wang H Zeng X Mo Y He B Lin H Lin J. Enterobactin-Specific Antibodies Induced by a Novel Enterobactin Conjugate Vaccine. Appl Environ Microbiol (2019) 85(10):e00358–19. doi: 10.1128/AEM.00358-19 PMC6498152 30877122

[9] Zeng X Wang H Huang C Logue CM Barbieri NL Nolan LK. Evaluation of the Immunogenic Response of a Novel Enterobactin Conjugate Vaccine in Chickens for the Production of Enterobactin-Specific Egg Yolk Antibodies. Front Immunol (2021) 12:629480. doi: 10.3389/fimmu.2021.629480 33868248 PMC8050339

[10] Akita E Nakai S. Comparison of Four Purification Methods for the Production of Immunoglobulins From Eggs Laid by Hens Immunized With an Enterotoxigenic *E. Coli* Strain. J Immunol Methods (1993) 160(2):207–14. doi: 10.1016/0022-1759(93)90179-B 8459107

[11] Pauly D Chacana PA Calzado EG Brembs B Schade R. IgY Technology: Extraction of Chicken Antibodies From Egg Yolk by Polyethylene Glycol (PEG) Precipitation. J Vis Exp (2011) 51:3084. doi: 10.3791/3084 PMC3197133 21559009

[12] Svihus B. Function of the Digestive System. J Appl Poult Res (2014) 23(2):306–14. doi: 10.3382/japr.2014-00937

[13] Kadhim KK Zuki A Noordin M Babjee S Zamri-Saad M. Activities of Amylase, Trypsin and Chymotrypsin of Pancreas and Small Intestinal Contents in the Red Jungle Fowl and Broiler Breed. Afr J Biotechnol (2011) 10(1):108–15. doi: 10.5897/AJB10.1553

[14] Ducarmon Q Zwittink R Hornung B Van Schaik W Young V Kuijper E. Gut Microbiota and Colonization Resistance Against Bacterial Enteric Infection. Microbiol Mol Biol Rev (2019) 83(3):e00007–19. doi: 10.1128/MMBR.00007-19 PMC6710460 31167904

[15] Shires A Thompson J Turner B Kennedy P Goh Y. Rate of Passage of Corn-Canola Meal and Corn-Soybean Meal Diets Through the Gastrointestinal Tract of Broiler and White Leghorn Chickens. Poult Sci (1987) 66(2):289–98. doi: 10.3382/ps.0660289 3588495

[16] Van der Klis J Verstegen M De Wit W. Absorption of Minerals and Retention Time of Dry Matter in the Gastrointestinal Tract of Broilers. Poult Sci (1990) 69(12):2185–94. doi: 10.3382/ps.0692185 2084676

[17] Bohak Z. Purification and Characterization of Chicken Pepsinogen and Chicken Pepsin. J Biol Chem (1969) 244(17):4638–48. doi: 10.1016/S0021-9258(18)93672-0 4897245

[18] Diraviyam T Zhao B Wang Y Schade R Michael A Zhang X. Effect of Chicken Egg Yolk Antibodies (IgY) Against Diarrhea in Domesticated Animals: A Systematic Review and Meta-Analysis. PLoS One (2014) 9(5):e97716. doi: 10.1371/journal.pone.0097716 24846286 PMC4028221

[19] Merchant HA McConnell EL Liu F Ramaswamy C Kulkarni RP Basit AW. Assessment of Gastrointestinal Ph, Fluid and Lymphoid Tissue in the Guinea Pig, Rabbit and Pig, and Implications for Their Use in Drug Development. Eur J Pharm Sci (2011) 42(1–2):3–10. doi: 10.1016/j.ejps.2010.09.019 20932902

[20] McConnell EL Basit AW Murdan S. Measurements of Rat and Mouse Gastrointestinal Ph, Fluid and Lymphoid Tissue, and Implications for *In Vivo* Experiments. J Pharm Pharmacol (2008) 60(1):63–70. doi: 10.1211/jpp.60.1.0008 18088506

[21] Beasley DE Koltz AM Lambert JE Fierer N Dunn RR. The Evolution of Stomach Acidity and Its Relevance to the Human Microbiome. PLoS One (2015) 10(7):e0134116. doi: 10.1371/journal.pone.0134116 26222383 PMC4519257

[22] Ash R. Acid Secretion by the Abomasum and its Relation to the Flow of Food Material in the Sheep. J Physiol (1961) 156(1):93–111. doi: 10.1113/jphysiol.1961.sp006660 13684719 PMC1359936

[23] Van Winden S Müller K Kuiper R Noordhuizen J. Studies on the pH Value of Abomasal Contents in Dairy Cows During the First 3 Weeks After Calving. J Vet Med A Physiol Pathol Clin Med (2002) 49(3):157–60. doi: 10.1046/j.1439-0442.2002.00429.x 12019957

[24] Kovacs-Nolan J Mine Y. Microencapsulation for the Gastric Passage and Controlled Intestinal Release of Immunoglobulin Y. J Immunol Methods (2005) 296(1–2):199–209. doi: 10.1016/j.jim.2004.11.017 15680164

[25] Li X-Y Jin L-J McAllister TA Stanford K Xu J-Y Lu Y-N. Chitosan-Alginate Microcapsules for Oral Delivery of Egg Yolk Immunoglobulin (IgY). J Agric Food Chem (2007) 55(8):2911–7. doi: 10.1021/jf062900q 17362028

[26] Wu X Zhao S Zhang J Wu P Peng C. Encapsulation of EV71-Specific IgY Antibodies by Multilayer Polypeptide Microcapsules and its Sustained Release for Inhibiting Enterovirus 71 Replication. RSC Adv (2014) 4(28):14603–12. doi: 10.1039/c3ra46943c

[27] Gu L McClements DJ Li J Su Y Yang Y Li J. Formulation of Alginate/Carrageenan Microgels to Encapsulate, Protect and Release Immunoglobulins: Egg Yolk IgY. Food Hydrocoll (2021) 112:106349. doi: 10.1016/j.foodhyd.2020.106349

